# Mental Health in the Era of the Second Wave of SARS-CoV-2: A Cross-Sectional Study Based on an Online Survey among Online Respondents in Poland

**DOI:** 10.3390/ijerph18052522

**Published:** 2021-03-04

**Authors:** Mateusz Babicki, Ilona Szewczykowska, Agnieszka Mastalerz-Migas

**Affiliations:** 1 Department of Family Medicine, Wroclaw Medical University, 51-141 Wroclaw, Poland; agnieszka.mastalerz-migas@umed.wroc.pl; 2 Intensive Care Unit, Wroclaw Medical University, Borowska Street 213, 50-556 Wrocław, Poland; ilo.sz@wp.pl

**Keywords:** mental health, second wave of pandemic, COVID-19, GHQ-28, anxiety

## Abstract

The main objective of this study was to assess mental health during the COVID-19 second wave. The study was conducted using a proprietary questionnaire that had been provided via the Internet to online respondents in Poland. The questionnaire questions included a socio-geographic assessment, proprietary questions assessing the respondents’ current approach to the COVID-19 pandemic, as well as a standardised psychometric tool—GHQ-28. The study involved 2155 respondents, 99.8% of whom gave their consent for the participation in the study. A mean GHQ score was 29.25 ± 14.94 points. The criterion for minor mental disorders (≥24 points) was met by 1272 (59.2%) respondents. In overall interpretation as well as in each of GHQ-28 subscales, women obtained significantly higher scores than men (*p* < 0.001). The restriction on earning opportunities during the COVID-19 pandemic is significantly associated with the feeling of anxiety/insomnia severity among the respondents (9.96 vs. 8.82 points; *p* < 0.001). The COVID-19 pandemic, although it has already been experienced for nearly a year, has had a significant association with the general mental health of the respondents in Poland. There is a strong need to implement special programs that offer psychological support in the era of the COVID-19 pandemic, especially for those who had direct experience with COVID-19 infection.

## 1. Introduction

In December 2019, viral pneumonia of previously unknown aetiology was first diagnosed in the Chinese city of Wuhan. Along with further research, a coronavirus pathogen, named SARS-CoV-2, was identified and the disease caused by it was specified as COVID-19. Due to the rapid spread of the pathogen worldwide, on 11 March 2020, WHO decided to declare the threat of a pandemic [1]. That situation has resulted in radical changes both in the everyday functioning of people and in research priorities—the search for effective means of prevention against SARS-CoV-2 became their main goal. [2]

The rapid changes over a short period, the uncertainty of the next day, and the fear of death may significantly affect human mental health. This can be confirmed by the previous experience with the MERS epidemic in 2012–2013 [3,4]. In addition to a direct impact on human physical health, the pandemic causes several indirect complications, including significant deterioration of mental health. It was proved that SARS-CoV-2 displayed the ability to directly affect the central nervous system (CNS), leading to the development of psychotic symptoms [5]. The measures preventing the spread of SAR-CoV-2 (in the form of social quarantine/home isolation) may result in the development of post-traumatic stress disorder (PTSD), an increase in anxiety-related symptoms, as well as depression severity, accompanied by a reduced sense of the quality of life [6]. There is evidence of effects on mental health long after the end of isolation [7]. Social distancing, which is currently one of the most effective methods of limiting the spread of SARS-CoV-2, has a significant impact on the deterioration of both mental and physical condition [8]. Many people develop defensive routines to avoid possible exposure to COVID-19. Mental condition might be adversely affected by limited meetings with friends and interpersonal interactions [9]. Moreover, loneliness, intensified by social isolation, is also considered an independent risk factor of suicide [10]. It should be noted that particularly in the case of young people interactions with the peer group, broadening one’s own autonomy and reducing ties with parents is crucial. At present, however, the opportunities to do so are significantly limited [11]. In many countries, including Poland, a kind of social rebellion against government restrictions and violation of applicable rules has been observed. This, in turn, increases the risk of SARS-CoV-2 transmission [12]. It has been found that at the time of the pandemic, people are faced with anxiety related to the uncertainty of the next day, fear of death, anxiety about the health of loved ones, as well as their own financial viability [13]. The impact of the COVID-19 pandemic on mental health is significantly linked to its devastating association with the global economy, which leads to an economic crisis and thus an increased risk of job loss and loss of livelihood [14]. This may result in an increase in suicides due to unemployment. Such an analysis was conducted in Canada, where the number of suicides is estimated to increase from 418 to 2114 [15]. Those results are consistent with what was observed in the USA, Pakistan, India, France, Italy, and Germany [16,17].

The incidence curve in Poland and other countries shows the tendency of SARS-CoV-2 to occur in “waves of infections” [18]. However, after nearly a year of restrictions, people slowly got used to living in a new reality. Every day we learn more about the virus, and immunization programme gives hope for a return to the normal life. Despite this, the persistent state of the pandemic still has an impact on the mental well-being of people.

Over the past year, many reports concerning the population mental health during the COVID-19 pandemic were published [19]. Currently, there are not many available reports assessing mental health during the second wave of the COVID-19 pandemic.

To sum up, there is an urgent need to pay more attention to human mental health during the COVID-19 pandemic. Systematic assessment and long-term observation of this issue is also of great importance. Despite the awareness of the limitations of this study, it aims to assess the current mental health of the respondents nearly 1 year after the first identification of COVID-19 worldwide. The objective of this paper was to assess how COVID-19-related preventive behaviours and the prevalence of minor mental disorders differed depending on demographic characteristics and experience with COVID-19. The following hypotheses were proposed: (1) The ongoing epidemiological situation has an impact on mental condition. (2) There is a difference in the prevalence of mental disorders and COVID-19 prevention attitudes depending on gender, location, and experience with COVID-19. (3) COVID-19 prevention attitudes and a sense of anxiety correlate with a probability of the occurrence of mental disorders.

## 2. Materials and Methods

### 2.1. Methods

This study was based on a proprietary questionnaire provided via the Internet at the peak of the second wave of the COVID-19 pandemic from 3 November 2020 to 29 November 2020. It was the time when the highest numbers of new cases per day (min. 19,152, max. 27,875 cases) and deaths due to COVID-19 (min. 92 max. 637 deaths) were found in Poland, which was also associated with the introduction of new legal restrictions [18]. Measures were introduced in the extension of a ban on stationary catering activities, take-away only. The activities of swimming pools, aquaparks, gyms, solariums, clubs, and fitness centres have been suspended, and sports events could take place only without the participation of the public. Remote learning for children was maintained and children under 16 were forbidden to leave their homes during the day without adult supervision. Commercial activities were limited and hotels were only available for business purposes.

The survey was fully anonymous and voluntary. It was distributed through social media and email. The respondents were Polish residents over the age of 18. Prior to the participation in the survey, the respondents were informed about the methodology and objectives of the study, as well as its estimated duration. Then they gave their informed written consent for the participation in the study. The respondents could withdraw from participation at any stage of completing the questionnaire, without providing reasons. The survey was conducted in Polish. The study was conducted in accordance with the principles of the Declaration of Helsinki (DoH).

The questionnaire comprised four sections, including a sociodemographic assessment (age, sex, place of residence, level of education, marital status), a subsection for healthcare professionals and related to experience with COVID-19 disease (exposure to SARS-CoV-2, being in quarantine, illness or death of a loved one), psychiatric history (psychiatric treatment in the past, the use of psychiatric drugs), and the need to use specialist services in connection with the ongoing COVID-19 pandemic. The respondents’ attitudes towards the COVID-19 pandemic were assessed, taking into account the assessment of government actions and compliance with the obligation to wear protective masks in open and closed spaces. The respondents’ approach to reducing meetings with friends and family as well as leaving home to a minimum was assessed. To assess the respondents’ attitude towards the COVID-19 pandemic, the participants to the study were asked to answer whether due to the COVID-19 pandemic they avoid meetings with their immediate family and friends and refrain from leaving home. In all cases, the answers are: Strongly agree/agree/neither yes nor no/disagree/strongly disagree. The level of anxiety of the respondents was assessed by the questions “On a scale of 1–10 (1—no anxiety, 10—extreme anxiety), how big is your fear of COVID-19 infection?” The second question was similar but it referred to the concern for a loved one.

The last part consist of standardized psychometric tool “Mental Health Assessment by D. Goldberg”—GHQ-28.

The 28-item General Health Questionnaire (GHQ-28) is a psychometric tool used for assessing minor mental disorders in the overall population. It is based on a four-point Likert scale (0—not at all, 1—no more than usual, 2—rather more than usual, 3—much more than usual). The maximum possible number of available points is 84. The cut-off point of clinical significance was 24 points [20]. Interpretation of the scale can also be done at the level of four subscales that included individual questions covering relevant somatic symptoms (items 1, 3, 4, 8, 12, 14, and 16), anxiety and insomnia (items 2, 7, 9, 13, 15, 17 and 18), social dysfunctions (items 5, 10, 11, 25, 26, 27 and 28), and severe depression (items 6, 19, 20, 21, 22, 23, and 24). [21] The original questionnaire is presented in the Supplementary material—Survey.

### 2.2. Statistical Analysis

The statistical analysis was conducted using Statistica software, version 13.3 (StatSoft, Hamburg, Germany).

The chi-squared test was used for comparing categorical variables. An analysis of interval scales was conducted at the level of basic descriptive statistics. The normality of distribution for the variables was evaluated by means of three different statistical tests: the Kolmogorov–Smirnov test, Lilliefors test, and Shapiro–Wilk W test. If variables did not meet the criterion of the normal distribution, the Mann–Whitney U or Kruskal–Wallis test was used.

The analysis of covariance (ANCOVA) was performed to investigate the differences in the levels of psychopathological manifestations between the groups after co-varying for potential confounding factors, such as age, sex, marital status, and level of education.

The GHQ-28 scale was analysed by assessing the prevalence of minor mental disorders in the COVID-19 era and factors that could affect it. Finally, the relationship between social constraints and the GHQ-28 scale was assessed.

The level of statistical significance was set at 0.05 at each statistics stage.

## 3. Results

### 3.1. Participants

A total of 2155 respondents were surveyed, of whom 0.2% (5) did not agree to participate in the study. Therefore, 2150 questionnaires were eligible for the study.

The mean age was 33.17 (min. 15; max. 75; SD 9.36). The vast majority were women—1759 (81.8%). A significant majority of the respondents had a university degree (71.4%) and lived in a city/town >250,000 population (59.8%). A detailed description of the study group was shown in Table 1.

### 3.2. Assessment of Social Attitudes towards COVID-19 Pandemic

Overall, 4.4% (94) of the respondents assessed the overall government response to the COVID-19 pandemic as good or very good, while 58.8% (1264) similarly assessed the COVID-19-related restrictions implemented in the country. The vast majority of respondents, i.e., 89.2% (1916), said they complied with the obligation to wear masks in open spaces (parks, playgrounds), and 98.28% (2113) of the respondents claimed that they wore masks indoors. During the second wave of the COVID-19 pandemic, the respondents limited their social activities, including meetings with family and friends, to varying degrees. A significantly higher percentage of the respondents agreed or strongly agreed that due to the COVID-19 pandemic they reduced meetings with friends (62.33%) rather than with family (55.22%). Additionally, 68.14% of the respondents agree/strongly agree when asked whether they limited leaving home to a minimum. Of the respondents, 61.8% searched for information regarding SARS-CoV-2 on a daily basis, and 60.9% viewed statistics on the number of COVID-19-related cases and deaths in the country. The detailed response distribution was shown in Table 2.

According to 565 respondents (26.27%), we should stay at home to prevent the COVID-19 pandemic, whereas 874 (40.65%) respondents were unable to answer that question and 708 (33.08%) did not agree with it.

In each of the aspects concerning the limitation of meetings with friends, family, as well as the limitation of leaving the house, women were much more likely to have a positive attitude than men. A similar correlation was observed in the case of respondents with higher education and those in whom the closest person died due to COVID-19 (*p* < 0.001). Detailed results were shown in Table 3 and Table 4.

In the linear analysis of the level of anxiety (from 1 to 10 points) related to contracting COVID-19, the mean value was 5.34 ± 2.62, and the most common answer was “7”. In the case of concern about COVID-19 infection of relatives and loved ones, the average was 7.86 ± 2.567 and the most common value was 10 points.

### 3.3. An Analysis of GHQ-28

In the overall analysis of GHQ-28, the mean score was 29.25 (SD 14.94; min.1; max 82) points. The criterion for minor mental disorders (≥24 points) was met by 1272 (59.2%) respondents.

A detailed analysis of the influence of individual factors on the GHQ-28 result is shown in Table 5. In overall interpretation, as well as in each of the GHQ-28 subscales, women obtained significantly higher scores than men (*p* < 0.001). Neither the level of education, place of residence, nor the marital status of the respondents had a statistically significant association with the mean scores in GHQ-28 (*p* > 0. 05). The restriction on earning opportunities during the COVID-19 pandemic was significantly associated with the feeling of anxiety/insomnia severity among the respondents (9.96 vs. 8.82; *p* < 0.001). The respondents who completely lost their income had a mean GHQ-28 score of 33.71 ± 15.67 points. With a loss of more than 25% of income, the score was 33.38 ±15.25. In those whose income value did not change at all, the score was 28.21 ± 14.69 (*p* < 0.001).

In the overall assessment, as well as in the subscale assessing somatic symptoms, anxiety and insomnia, healthcare professionals show higher scores than non-medical workers (*p* < 0.001).

According to the survey, the 7.3% of the respondents who availed of psychiatrist or psychologist services during the COVID-19 pandemic had higher mean GHQ-28 score than those who did not receive psychological help (*p* < 0.001). Their scores were significantly higher in each of the analysed subscales (*p* < 0.001). As in the case of past psychiatric treatment and the use of psychiatric medications (*p* < 0.001). The respondents with a history of chronic conditions scored 3.67 points more than healthy individuals (*p* < 0.001).

Individual experience with COVID-19, resulting from one’s own illness, illness of a family member/friend, and death among loved ones, were associated with the final score of GHQ-28 (*p* < 0.05). The respondents who were under isolation while participating in the study scored 33. 67 ± 14.51 points compared to convalescents who scored 29.01 ± 14.87 points (*p* < 0.001). Those respondents also had much more aggravation of somatic symptoms, anxiety, and insomnia than convalescents and individuals without confirmed COVID-19 infection (*p* < 0.001).

Both information retrieval and tracking of statistics concerning COVID-19-related cases/deaths was associated with the overall assessment of GHQ-28 and subscales assessing somatic disorders, anxiety/insomnia (*p* < 0.001).

The ANCOVA covariance analysis showed a statistically significant effect of gender on the overall GHQ-28 score in the field of psychiatric treatment in the past (F = 6.366; *p* = 0.011). In the GHQ-28 subscales, the gender effect was statistically significant in the subscale evaluating anxiety symptoms in health care workers (F = 5.115; *p* = 0.023), psychiatric past (F = 10.43; *p* = 0.002), psychiatric drugs (F = 8.428; *p* = 0.003), and quarantine (F = 3.279; *p* = 0.037). In other cases, the significance was not demonstrated.

The relationship between limited meetings with family and friends and minimized trips out of the house, anxiety about one’s own health, concern for health of the loved ones, and the GHQ28 scores is shown in Table 6.

## 4. Discussion

In this study, 1272 (59.2%) respondents obtained a GHQ-28 score ≥24 points, which indicates the presence of minor mental disorders. The mean GHQ-28 score was 29.25 ± 14.94 points. It was noticed that women, people with limited earning potential, people using specialist services, and those suffering from chronic diseases, as well as those who search for information about COVID-19 and follow daily statistics, obtained significantly higher results. In the assessment of attitudes towards the COVID-19 pandemic, the vast majority of respondents agree/strongly agree that due to the pandemic there are limited meetings with family (53.22%), friends (62.33%) and minimized trips out of the house (68.14%). In each case, women, people with higher education, and respondents whose relatives were confirmed with COVID-19 or died from the disease show a reduction in interpersonal contacts. A positive correlation was observed between limited contacts with family and friends, minimized trips out of the house, and the subjective fear of one’s own and loved ones’ illness, and the GHQ-28 scale.

In the period before the pandemic, mental health of Poles was examined as part of the 3-year EZOP project, which ended in 2012. Project results showed that 23.7% of the respondents were diagnosed with at least one mental illness based on ICD-10 and DSM-IV. Neurotic disorders were observed in 10% of the respondents and mood disorders in 3.5% of the respondents [22]. According to the report published in 2019, the European Union mental health report concerning the diagnosis of anxiety disorders in doctor’s certificates issued in Polish certificate is 3.9% and the depressive certificate is 5.1%, based on a WHO study [23].

Compared to reports from Poland during the first wave of the COVID-19 pandemic (when COVID-19-related cases and deaths were significantly lower), the study involving 443 individuals showed a mean GHQ-28 score of 31.74 ± 16.93 [24]. The data obtained from India show a risk of mental disorders in nearly 42.16% of respondents with a mean GHQ-28 score of 24.18 ± 14.00 [25]. In Fars, a province of Iran, 46.1% of respondents included in the survey had a GHQ-28 score indicating a risk of mental disorders [26]. Meta-analyses of studies assessing the impact of the COVID-19 pandemic on mental health clearly show that it has a tremendous association with population health: an increase in social anxiety, severity of depressive symptoms, and risk of developing PTSD. The main influencing factors include female sex, a low level of education, and the coexistence of chronic diseases [27]. A study conducted in 2017 and 2020 in the Czech Republic, which is Poland’s closest neighbour, showed an increase in mental disorder control from 20.02% to 29.63%, showing the impact of the COVID-19 pandemic and the numerous restrictions associated with it [28]. Comparing the results of our study with the results of other studies published before the pandemic (despite a different methodology and study groups), it can be supposed that COVID-19 pandemic has had a significant impact on the mental condition of Polish respondents [13,22,23,24]. In both this study and worldwide reports, women are significantly more likely to show a tendency towards mental disorders in response to a stressful situation, and the significant prevalence of women (81.8%) may significantly affect the final outcome of the study’s analysis. However, most studies in the form of online surveys have a female preponderance due to greater willingness and inclination of women to participate in surveys [13,24,25,26].

The results of this study are consistent with global reports assessing the enormous impact of mass media on creating attitudes of people. Both daily tracking of statistics reporting the number of new COVID-19-related cases and deaths and the web search for COVID-19-related information is closely related to the final score of the GHQ-28 scale in whole and its subscales. This result correlates with a study conducted in Wuhan, in which information retrieval was an independent predictor of an increased feeling of anxiety and depression [27]. A definite increase in searches for COVID-19-related information among online users is also of note [29,30,31]. It should also be noted that mass media can be a powerful tool to improve the mental conditions of people by increasing awareness of mental disorders as well as conveying information on appropriate mental support programs. However, it cannot be unequivocally stated that searching for information and tracking statistics of COVID-19 adversely affects mental condition as the study did not exclude the phenomenon of reverse causality.

According to this study, 848 respondents (39.2%) were active healthcare professionals, in whom both the aggravation of somatic, anxiety-related symptoms, insomnia severity, and the overall analysis of GHQ-28 are significantly higher than in non-medical workers. These findings are consistent with global reports, which clearly show significant psychological strain in medical personnel, especially when there is limited access to personal protective equipment [31,32,33,34].

The study revealed different respondents’ attitudes towards the assessment of the level of anxiety about their own health and the health of their loved ones; the respondents were definitely more concerned about their family members and close friends than about themselves. The severity of anxiety was correlated with the risk of developing mental disorders [35]. In this study, the mean score for the assessment of the anxiety about one’s own health (the scale ranged from 1 to 10 points) was 5.34 compared to 7.85 for the anxiety about the health of loved ones. A similar analysis conducted during the first wave of the COVID-19 pandemic showed that the level of anxiety about one’s own health was 5.50 points [13].

The authors are aware of the limitations to this study, which undoubtedly include the method of data collection, via an online questionnaire. In the current epidemiological situation, however, this type of data collection is a safe and validated research method. According to reports, the advantages of this research method are significantly lower levels of stress and better psychological comfort of individuals who complete anonymous online questionnaires [36,37,38]. On the other hand, a study of the self-esteem type is burdened with the risk of error resulting from the bias of the respondents, the possibility of misinterpretation of questions, and the lack of honesty and inclination to provide more acceptable answers [39]. Another methodological limitation of this study is the lack of possibility to assess both the number of questionnaires that were not completed at any stage of the study and the number of individuals reached by the survey. A current literature review suggests that individuals who refuse to participate in surveys may be more likely to suffer from mental health conditions [40,41]. The study does not provide any definite diagnosis due to the use of only the GHQ-28 questionnaire. It should also be noted that the group presented in this study is not representative of Polish society. The vast majority of women, people with higher education and those living in the city >250,000 population, were identified as potential confounders. The ANCOVA analysis of covariance showed that these variables had a different influence on the final score of the GHQ-28 scale and its components. The level of education significantly influenced the differences in responses between medical and non-medical workers. The predominance of women had an impact on the overall anxiety subscale score and in relation to profession, psychiatric past, and experience with COVID-19. Therefore, to assess the Polish population, it is necessary to perform a study with a representative study group.

To sum it up, this study indicates the persistence of high risk of developing mental disorders among the society in response to the current epidemiological situation, the uncertainty of the next day, and the anxiety about life and health of themselves and loved ones. Based on the experience of the epidemic and current reports, it is necessary to develop appropriate mental support tools for the most needed and active activities propagated through the mass media. There is a need for long-term studies using representative samples of populations to fully understand the societal impact of SARS-CoV-2 on specific populations.

## 5. Conclusions

1. The COVID-19 pandemic heavily influences the respondents’ decline in mental health.
2. There is a need to implement appropriate psychological support programs, especially for those who recovered from COVID-19 or lost their loved one due to the disease.
3. The loss of professional stability, in the form of a job loss and a salary reduction, has a significant impact on mental health, especially in the era of the COVID-19 pandemic.

## Figures and Tables

**Table 1:** Description of the study group.

Variable		Value (*n* (%); M, SD)
Sex	Male	391 (18.2%)
	Female	1759 (81.8%)
Age		33.17 ± 9.36
Place of residence	city/town >250,000 population	1286 (59.8%)
	city/town >250,000–50,000 population	327 (15.2%)
	city/town of up to 50,000 population	238 (11.1%)
	countryside	299 (13.9%)
Level of education	higher (university degree)	1535 (71.4%)
	incomplete higher	285 (13.3%)
	secondary	292 (13.5%)
	vocational	9 (0.4%)
	lower secondary	19 (0.9%)
	primary	10 (0.5%)
Marital status	married	1025(47.7%)
	in a romantic relationship	506 (23.5%)
	divorced	75 (3.5%)
	widowed	19(0.9%)
	solitude	524 (24.4%)
Restriction on earning opportunities	Yes, I lost my job	97 (4.5%)
	Yes, a decrease in income ≥25%	132 (6.1%)
	Yes, a decrease in income ≤25%	118 (5.5%)
	Yes, income has remained unchanged	130 (6.1%)
	No	1456 (67.7%)
	I didn’t work before or during the pandemic	217 (10.1%)
Healthcare professional	Yes	848 (39.4%)
	No	1302 (60.6%)
Use of psychiatrist/psychologist services due to the COVID-19 pandemic	Yes	157 (7.3%)
	No	1993 (92.7%)
Use of psychiatric medications	Yes	383 (17.8%)
	No	1767 (82.2%)
Past psychiatric treatment	Yes	417 (19.4%)
	No	1733 (80.6%)
Chronic conditions, e.g., heart disease, lung disease	Yes	486 (22.6%)
	No	1664 (77.4%)
Being under quarantine	Yes, I am under quarantine	98(4.5%)
	Yes, I was under quarantine	324 (15.1%)
	No	1728 (80.4%)
Recovering from COVID-19	Yes, I’m sick now	81 (3.8%)
	Yes, I recovered from COVID-19	206 (9.7%)
	No	1863 (86.7%)
COVID-19 disease confirmed in a family member/close friend	Yes	1483 (68.9%
	No	667 (31.1%)
COVID-19-related death	Yes, confirmed in a family member	88 (4.1%)
	Yes, confirmed in a close friend	222 (10.3%)
	No	1840 (85.6%)

**Table 2:** Distribution of responses assessing COVID-19-related social restrictions.

Answer	Limited Meetings with Family	Limited Meetings with Friends	Minimized Trips out of the House
	*n* = 2150 (%)		
**I strongly agree**	19.68	25.86	30.93
**I agree**	35.54	36.47	37.21
**I don’t agree or disagree.**	12.88	10.04	6.74
**I disagree**	20.60	17.16	15.95
**I strongly disagree**	11.30	10.47	9.17
	Searching information about COVID-19		Tracking the statistics about COVID-19
**Yes**	61.8		60.9
**No**	38.2		39.1

**Table 3:** Limitation of social encounters by sex, place of residence, level of education, marital status, and restrictions on earning opportunities.

Variable		Limited Meetings with Family *n* = 2150 (%)		Limited Meetings with Friends *n* = 2150 (%)		Minimized Trips out of the House *n* = 2150 (%)		The Need to Stay at Home to Prevent the Pandemic *n* = 2150 (%)	
		Yes *	*p*	Yes *	*p*	Yes *	*p*	Yes	*p*
**Sex**	Male	50.64	0.078	50.9	<0.001	58.31	<0.001	22.31	<0.001
	Female	56.22		64.87		70.33		27.21	
**Place of residence**	city/town >250,000 population	58.48	<0.001	63.81	0.463	68.75	0.159	26.95	0.495
	city/town >250,000–50,000 population	58.35		63.61		69.73		27.52	
	city/town of up to 50,000 population	49.16		59.67		64.71		21.43	
	countryside	45.82		57.52		66.55		26.17	
**Level of education**	higher (university degree)	61.57	<0.001	69.30	<0.001	73.36	<0.001	28.77	<0.001
	incomplete higher	46.67		51.58		62.46		24.91	
	secondary	32.59		40.41		50.34		17.18	
	vocational	22.22		26.32		42.11		10.53	
	lower secondary	15.79		22.22		22.22		0	
	primary	20		30		40		10	
**Marital status**	married	59.03	0.086	68	<0.001	72.87	<0.001	28.61	0.081
	in a romantic relationship	51.68		58.22		63.37		25.01	
	divorced	53.33		56		62.66		20	
	widowed	63.16		78		94.74		26.32	
	solitude	51.33		55.73		63.55		24.09	
**Restriction on earning opportunities**	Yes, I lost my job	41.23	<0.001	47.43	<0.001	52.58	<0.001	20.62	<0.001
	Yes, a decrease in income ≥25%	55.94		61.37		63.62		27.27	
	Yes, a decrease in income ≤25%	53.79		60.17		63.56		24.58	
	Yes, income has remained unchanged	50.00		56.93		63.07		15.38	
	No	58.38		65.05		71.29		28.08	
	I didn’t work before or during the pandemic	43.78		55.76		62.21		23.96	

* Answers: I strongly agree/I agree.

**Table 4:** Limitations of social encounters in terms of psychiatric history, the burden of chronic conditions, and experience with COVID-19.

Variable		Limited Meetings with Family *n* = 2150 (%)		Limited Meetings with Friends *n* = 2150 (%)		Minimized Trips out of the House *n* = 2150 (%)		The Need to Stay at Home to Prevent the Pandemic *n* = 2150 (%)	
		Yes *	*p*	Yes *	*p*	Yes *	*p*	Yes	*p*
**Use of psychiatrist/psychologist services due to the COVID-19 pandemic**	Yes	66.24	0.019	70.07	0.248	68.79	0.593	28.03	0.593
	No	54.34		61.72		68.09		26.18	
**Use of psychiatric medications**	Yes	61.88	0.016	66.32	0.365	72.06	0.178	25.66	0.306
	No	53.77		61.46		67.29		26.47	
**Past psychiatric treatment**	Yes	57.08	0.094	64.27	0.546	68.82	0.005	28.72	0.167
	No	54.77		61.85		67.97		25.79	
**Chronic conditions, e.g., heart disease, lung disease**	Yes	58.84	0.028	65.84	0.015	73.87	0.013	28.45	0.417
	No	54.15		61.30		66.47		25.69	
**Being under quarantine**	Yes, I am under quarantine	64.29	0.264	68.37	0.366	77.56	0.088	35.71	0.001
	Yes, I was under quarantine	57.10		66.36		71.91		25.31	
	No	54.34		61.22		66.90		25.97	
**Recovering from COVID-19**	Yes, I’m sick now	70.37	0.050	76.55	0.206	80.25	0.033	38.27	0.002
	Yes, I recovered from COVID-19	58.25		66.51		71.35		23.79	
	No	54.21		64.25		67.22		26.08	
**COVID-19 disease confirmed in a family member/close friend**	Yes	58.93	<0.001	65.14	<0.001	71.55	<0.001	28.16	<0.001
	No	46.93		56.07		60.57		22.22	
**COVID-19-related death**	Yes, confirmed in a family member	67.04	<0.001	72.72	<0.001	80.68	<0.001	36.36	<0.001
	Yes, confirmed in a close friend	67.12		74.77		83.78		39.64	
	No	53.21		60.32		65.06		24.22	

* Answers: I strongly agree /I agree.

**Table 5:** A detailed analysis of the effect of individual factors on GHQ-28 score and its subscales.

Variable (*n* = 2150)		GHQ-28		GHQ-28: Somatic Symptoms		GHQ-28: Anxiety/Sleep Disorder		GHQ-28: Social Dysfunctions		GHQ-28: Depression	
		M (SD)	*p*	M (SD)	*p*	M (SD)	*p*	M (SD)	*p*	M (SD)	*p*
Total sample		29.25 (14.94)		7.15 (4.32)		8.98 (3.53)		9.07 (5.24)		4.06 (4.54)	
Sex	Male	24.11 (12.94)	<0.001	5.58 (3.65)	<0.001	6.90 (4.66)	<0.001	8.13 (3.10)	<0.001	3.49 (4.09)	<0.001
	Female	30.4 (15.11)		7.49 (4.38)		9.55 (5.24)		9.17 (3.59)		4.18 (4.68)	
Place of residence	city/town >250,000 population	26.69 (15.11)	0.14	7.18 (4.27)	0.055	9.22 (5.21)	0.398	9.10 (3.62)	0.006	4.18 (4.67)	0.499
	city/town 250,000–50,000 population	30.13 (14.79)		7.50 (4.42)		9.33 (5.24)		9.15 (3.49)		4.13 (4.47)	
	city/town of up to 50,000 population	29.05 (15.13)		7.32 (4.69)		8.90 (5.45)		8.91 (3.43)		3.92 (4.68)	
	countryside	26.58 (13.89)		6.46 (4.08)		8.26 (5.10)		8.31 (3.17)		3.55 (4.18)	
Level of education	higher (university degree)	29.32 (14.47)	0.217	7.33 (4.33)	0.005	9.27 (5.17)	0.002	8.89 (3.40)	0.71	3.81 (4.21)	0.22
	incomplete higher	29.25 (14.95)		6.75 (3.92)		8.72 (5.11)		9.205 (3.85)		4.52 (4.78)	
	secondary	29.25 (17.34)		6.76 (4.59)		8.51 (5.68)		9.17 (3.86)		4.81 (5.63)	
	vocational	20.67 (12.42)		3.67 (1.87)		5.11 (3.79)		8.22 (3.56)		3.67 (4.94)	
	lower secondary	25.37 (10.62)		6.00 (4.03)		7.47 (4.21)		8.68 (2.69)		3.21 (2.57)	
	primary	34.70 (18.11)		6.90 (3.41)		9.60 (5.72)		10.50 (4.45)		7.70 (7.68)	
Marital status	married	27.73 (13.86)	0.071	7.13 (4.30)	0.702	8.95 (5.15)	0.130	8.56 (3.21)	<0.001	3.09 (3.75)	<0.001
	in a romantic relationship	31.14 (16.18)		7.38 (4.40)		9.55 (5.50)		9.40 (3.72)		3.09 (3.75)	
	divorced	27.15 (13.50)		7.38 (4.40)		9.55 (5.50)		9.40 (3.72)		4.81 (5.20)	
	widowed	32.00 (13.90)		7.26 (3.63)		9.89 (4.82)		10. (3.63)		4.84 (4.49)	
	solitude	30.64 (15.64)		6.99 (4.30)		8.88 (5.17)		9.45 (3.85)		5.31 (5.13)	
Restriction on earning opportunities	Yes, I lost my job	33.71 (15.67)	<0.001	7.22 (4.16)	0.161	10.09 (5.33)	<0.001	10.84 (4.00)	<0.001	5.57 (4.84)	<0.001
	Yes, a decrease in income ≥25%	33.37 (15.25)		7.88 (4.53)		10.56 (5.21)		10.25 (3.82)		4.70 (4.97)	
	Yes, a decrease in income ≤25%	32.23 (16.57)		7.87 (4.59)		10.19 (5.60)		9.36 (3.48)		4.81 (5.09)	
	Yes, income has remained unchanged	28.22 (14.70)		7.04 (4.13)		9.18 (5.14)		9.02 (5.25)		5.85 (5.39)	
	No	28.01 (14.38)		7.03 (4.32)		8.76 (5.19)		8.61 (3.32)		3.60 (4.27)	
	I didn’t work before or during the pandemic	32.14 (15.98)		7.12 (4.20)		9.18 (5.14)		10.0 (3.89)		5.85 (5.39)	
Psychiatrist/ psychologist services during the pandemic	Yes	41.59 (15.85)	<0.001	10.31 (4.61)	<0.001	13.03 (4.78)	<0.001	11.00 (3.85)	<0.001	7.24 (5.85)	<0.001
	No	28.28 (14.25)		6.89 (4.20)		8.76 (5.15)		8.83 (3.46)		3.80 (4.38)	
The use of psychiatric medications	Yes	34.91 (16.14)	<0.001	8.83 (4.59)	<0.001	10.49 (5.14)	<0.001	9.67 (3.91)	<0.001	5.92 (5.48)	<0.001
	No	28.03 (14.38)		6.78 (4.18)		8.77 (5.21)		8.83 (3.42)		3.65 (4.27)	
Healthcare professional	Yes	30.34 (14.89)	<0.001	7.83 (4.39)	<0.001	9.84 (5.25)	<0.001	8.86 (3.40)	0.667	3.81 (4.36)	0.131
	No	28.55 (15.16)		6.70 (4.21)		8.56 (5.17)		9.06 (3.61)		4.21 (4.76)	
Past psychiatric treatment	Yes	34.74 (16.02)	<0.001	8.59 (4.53)	<0.001	10.36 (5.12)	<0.001	9.76 (3.95)	<0.001	6.01 (5.49)	<0.001
	No	27.93 (14.36)		6.80 (4.19)		8.75 (5.22)		8.79 (3.39)		3.58 (4.20)	
Chronic conditions, e.g., heart disease, lung disease	Yes	32.10 (15.51)	<0.001	8.20 (4.41)	<0.001	9.79 (5.31)	<0.001	9.55 (3.67)	<0.001	4.54 (4.77)	<0.001
	No	28.43 (14.66)		6.84 (4.22)		8.86 (5.20)		8.81 (3.47)		3.91 (4.52)	
Being under quarantine	Yes, I am under quarantine	30.90 (15.07)	0.146	9.13 (4.68)	<0.001	9.14 (5.60)	0.497	9.37 (3.37)	0.203	3.27 (4.05)	0.013
	Yes, I was under quarantine	28.17 (14.49)		7.10 (4.32)		8.64 (5.27)		8.85 (3.32)		3.57 (4.30)	
	No	29.37 (15.01)		7.04 (4.27)		9.15 (5.21)		8.98 (3.58)		4.19 (4.66)	
Recovering from COVID-19	Yes, I’m undergoing recovery from COVID-19	33.67 (14.51)	0.061	10.91 (4.71)	<0.001	9.83 (5.84)	0.296	9.72 (3.34)	0.012	3.20 (3.46)	0.006
	Yes, I recovered from COVID-19	29.01 (14.87)		7.69 (4.44)		9.07 (5.46)		8.93 (3.46)		3.31 (4.22)	
	No	29.09 (14.94)		6.92 (4.21)		9.03 (5.19)		8.96 (3.54)		4.18 (4.66)	
COVID-19 confirmed in a family member/ close friend	Yes	29.78 (14.66)	0.002	7.41 (4.30)	<0.001	9.30 (5.14)	<0.001	9.09 (3.54)	0.011	3.96 (4.45)	0.901
	No	28.09 (15.49)		6.56 (4.31)		8.54 (5.41)		8.72 (3.47)		4.25 (4.86)	
COVID-19-related death	Yes, a family member	32.68 (15.45)	<0.001	8.45 (4.61)	<0.001	9.98 (5.19)	0.014	9.80 (3.39)	0.004	5.45 (5.25)	0.004
	Yes, in a close friend	30.56 (14.75)		7.96 (4.40)		10.07 (5.17)		9.05 (3.65)		3.48 (4.19)	
	No	28.89 (14.90)		6.99 (4.27)		8.90 (5.23)		8.93 (3.52)		4.06 (4.58)	
Information retrieval	Yes	30.46 (14.74)	<0.001	7.66 (4.35)	<0.001	9.66 (5.14)	<0.001	9.09 (3.52)	<0.001	4.03 (4.52)	0.593
	No	27.31 (15.06)		6.32 (4.12)		8.10 (5.26)		8.79 (3.54)		4.09 (4.68)	
Statistics tracking	Yes (60.9)	30.12 (14.85)	<0.001	7.51 (4.35)	<0.001	9.35 (5.19)	<0.001	9.06 (3.57)	0.006	4.09 (4.51)	0.008
	No	27.77 (14.95)		6.57 (4.21)		8.34 (5.24)		8.84 (3.46)		3.99 (4.69)	

**Table 6:** The relationship between limited meetings with family and friends and minimized trips out of the house; anxiety about one’s own health; concern for health of the loved ones; and the GHQ28 scores.

Variable (*n* = 2150)		GHQ-28	GHQ-28: Somatic Symptoms			GHQ-28: Anxiety/Sleep Disorder		GHQ-28: Social Dysfunctions		GHQ-28: Depression	
		M /R (SD)	*p*	M/R (SD)	*p*	M/R (SD)	*p*	M/R (SD)	*p*	M/R (SD)	*p*
Limited meetings with family	Strongly agree	32.70 (15.77)	<0.0001	8.41 (4.66)	<0.0001	10.49 (5.50)	<0.0001	9.36 (3.76)	0.002	4.44 (4.72)	0.027
	Agree	30.72 (14.67)		7.75 (4.22)		9.66 (5.10)		9.09 (3.56)		4.23 (4.61)	
	Neither yes nor no	28.55 (15.26)		6.75 (4.13)		8.36 (4.94)		9.08 (3.61)		4.35 (4.94)	
	Disagree	25.72 (13.02)		6.04 (3.95)		7.93 (4.82)		8.45 (3.12)		3.29 (3.93)	
	Strongly disagree	25.95 (15.23)		5.55 (3.88)		7.63 (5.40)		8.85 (3.53)		3.92 (4.84)	
Limited meetings with friends	Strongly agree	31.84 (15.21)	<0.0001	8.30 (4.56)	<0.0001	10.19 (5.31)	<0.0001	9.20 (3.75)	0.364	4.14 (4.50)	0.229
	Agree	29.79 (14.82)		7.42 (4.21)		9.38 (5.13)		8.96 (3.50)		4.03 (4.53)	
	Neither yes nor no	28.56 (14.81)		6.66 (4.17)		8.68 (5.05)		9.02 (3/54)		4.19 (4.56)	
	Disagree	26.41 (13.55)		6.06 (3.92)		7.86 (4.89)		8.73 (3.18)		3.75 (4.51)	
	Strongly disagree	26.37 (13.55)		5.60 (3.97)		7.56 (5.56)		8.87 (3.60)		4.34 (5.08)	
Minimized trips out of the house	Strongly agree	31.42 (15.39)	<0.0001	8.17 (4.61)	<0.0001	10.02 (5.35)	<0.0001	9.17 (3.79)	0.0006	4.06 (4.46)	0.008
	Agree	29.97 (14.47)		7.45 (4.05)		9.24 (9.99)		9.10 (3.46)		4.18 (4.55)	
	Neither yes nor no	29.03 (15.47)		6.53 (4.22)		9.10 (5.47)		9.35 (3.38)		4.04 (5.01)	
	Disagree	25.74 (13.47)		5.78 (3.83)		7.75 (4.93)		8.45 (3.20)		3.77 (4.51)	
	Strongly disagree	25.38 (15.63)		5.35 (4.08)		7.41 (5.42)		8.55 (3.46)		4.08 (4.98)	
		R	*p*	R	*p*	R	*p*	R	*p*	R	*p*
Anxiety about one’s own health		0.244	<0.001	0.274	<0.001	0.258	<0.001	0.110	<0.001	0.075	<0.001
Anxiety about health of loved ones		0.215	<0.001	0.284	<0.001	0.270	<0.001	0.135	<0.001	0.124	<0.001

## Data Availability

The data presented in this study are available on request from the corresponding author.

## Supplementary Materials

The following is available online at https://www.mdpi.com/1660-4601/18/5/2522/s1. Survey.
