# Supplementary material for: Mental Health in the Era of the Second Wave of SARS-CoV-2: A Cross-Sectional Study Based on an Online Survey among Online Respondents in Poland
Source: Int J Environ Res Public Health. 2021 Mar 4;18(5):2522. doi: 10.3390/ijerph18052522 (PMC7967546; doi:10.3390/ijerph18052522)
Supplement: Supplementary file 1 [file ijerph-18-02522-s001.pdf]

We would like to learn about your approach to the COVID-19 pandemic and assessment of the implemented "red zone" procedures throughout the country and, at the same time, to assess its impact on the mental condition.

The survey is entirely anonymous and voluntary. You can quit it at any stage.
It takes approximately 7 minutes.
It is addressed to all persons over 18 who live in Poland.

1. Do you agree to participate in the study?
    a) Yes
    b) No

Sociodemographic section

1. Sex: Female [ ] Male [ ]
2. Age: ….. [years]
3. Place of residence:
    a) Countryside
    b) City/town with a population under 50,000
    c) City/town with a population between 50,000 and 250,000
    d) City/town with a population over 250,000
4. Level of education:
    a) Primary
    b) Lower secondary
    c) Vocational
    d) Secondary
    e) Incomplete higher
    f) Higher (university degree)
5. Marital status:
    a) Married
    b) Divorced
    c) Widowed
    d) In a relationship
    e) Single
6. Are you a healthcare professional?
    a) Yes
    b) No
7. Have you been deprived of earning opportunities as a result of the pandemic?
    a) Yes, but I have already returned to work and earn the same amount as before
    b) Yes, I have already returned to work, but I earn up to 25% less than before
    c) Yes, I have already returned to work, but I earn over 25% less than before
    d) Yes, I have no income source
    e) No
    f) I have not worked before or during the pandemic
8. Have you used psychiatrist/psychologist support due to the pandemic?
    a) Yes
    b) No
9. Do you suffer from chronic diseases (i.e. hypertension, heart conditions, asthma etc.)
    a) Yes
    b) No
10. Have you been treated psychiatrically in the past?

a) Yes
b) No

11. If yes, for what reason?

Depression / Anxiety disorders / Sleep disorders / Behavioral disorders / Schizophrenia spectrum disorders / Addictions

12. Have you ever taken psychiatric medications (i.e. Ketrel, Alprazolam, Pramolan, etc.)

a) Yes
b) No

13. Have you been placed under quarantine by a Sanitary and Epidemiological Station during the COVID-19 pandemic?

a) Yes, I am now in quarantine
b) Yes, I have
c) No

14. Have you been diagnosed with COVID-19?

a) Yes, I am sick now
b) Yes, I have recovered from COVID-19
c) No

15. If so, what was the course of the disease?

a) Asymptomatic
b) Mild (e.g. headache, cough, temperature below 38 degrees Celsius, self-isolated)
c) Moderate (temperature above 38 degrees Celsius, dyspnoea, no hospitalization)
d) Hospitalization

16. Do you think the COVID-19 pandemic has affected your overall mental health?

a) Yes, it has significantly deteriorated
b) Yes, it has deteriorated
c) No
d) Yes, it has improved

Medical Professionals

1. If yes, what is your occupation:

Doctor / Dentist / Nurse / Paramedic / Laboratory diagnostician / Technician / Other

2. Have you been delegated to work at a COVID-19 ward by the hospital or state authorities?

a) Yes, by state authorities
b) Yes, by hospital authorities
c) No

3. Have you had direct contact with COVID-19 patients?

a) Yes, in a dedicated COVID-19 hospital
b) Yes, at a COVID-19 ward in a different than dedicated COVID-19 hospital
c) Yes, both in hospital and as a general practitioner
d) Yes, as a general practitioner
e) No

Attitudes

1. In your opinion, the state authorities' overall response to COVID-19 is?
   a) Very good
   b) Good
   c) Neither good nor bad
   d) Bad
   e) Very bad
2. Do you agree with the restrictions implemented (the whole country being the so-called "red zone") to fight the COVID-19 pandemic?
   a) Strongly agree
   b) Agree
   c) Neither agree nor disagree
   d) Disagree
   e) Strongly disagree
3. Since the amendment of recommendations regarding the obligation to wear masks, have you worn masks in open spaces, e.g. parks, playgrounds?
   a) Yes, always
   b) Yes, usually
   c) No
4. Since the amendment of recommendations regarding the obligation to wear masks, have you worn masks indoors, e.g. shopping centres, public transport vehicles?
   a) Yes, always
   b) Yes, usually
   c) No
5. Do you agree that you have been avoiding meetings with your family due to the COVID-19 pandemic?
   a) Strongly agree
   b) Agree
   c) Neither agree nor disagree
   d) Disagree
   e) Strongly disagree
6. Do you agree that you have been avoiding meetings with your friends due to the COVID-19 pandemic?
   a) Strongly agree
   b) Agree
   c) Neither yes nor no
   d) Disagree
   e) Strongly disagree
7. Do you agree that you have reduced leaving the house to the minimum (shopping, work etc.) due to the COVID-19 pandemic?
   a) Strongly agree
   b) Agree
   c) Neither agree nor disagree
   d) Disagree
   e) Strongly disagree
8. Are you afraid of getting infected with COVID-19?
   a) Yes, the same as other illnesses (i.e. heart diseases)
   b) Yes, but less than other illnesses (i.e. heart diseases)
   c) Yes, but more than other illnesses (i.e. heart diseases)
   d) Not at all
9. On a scale of 1 to 10 (1 - no anxiety, 10 - extreme anxiety), how significant is your anxiety regarding getting infected with COVID-19?……..

10. On a scale of 1 to 10 (1 - no anxiety, 10 - extreme anxiety), how significant is your anxiety regarding your loved ones being infected with COVID-19?……..

11. Do you search for additional information about COVID-19, e.g. online?
    a) Yes
    b) No
12. Do you follow daily statistics concerning COVID-19 infections and fatalities?
    a) Yes
    b) No
13. Should we all stay at home to contain the COVID-19 pandemic?
    a) Yes
    b) No
    c) It is hard to tell

The General Health Questionnaire (GHQ-28)

Have you recently:

1 GHQ1	**Been feeling well and enjoying good health?**	More than usual	Same as usual	Less than usual	Much less than usual
2 GHQ2	**Felt constantly under pressure?**	Not at all	No more than usual	Somewhat more than usual	Much more than usual
3 GHQ3	**Been experiencing a feeling of tightness or pressure in your head**	Not at all	No more than usual	Somewhat more than usual	Much more than usual
4 GHQ4	**Been getting headaches?**	Not at all	No more than usual	Somewhat more than usual	Much more than usual
5 GHQ5	**Felt capable of making decisions about different matters?**	More than usual	Same as usual	Less than usual	Incapable
6 GHQ6	**Found at times that you cannot do anything due to anxiety**	Not at all	No more than usual	Somewhat more than usual	Much more than usual
7 GHQ7	**Been irritable and/or in a bad mood?**	Not at all	No more than usual	Somewhat more than usual	Much more than usual
8 GHQ8	**Been feeling ill?**	Not at all	No more than usual	Somewhat more than usual	Much more than usual
9 GHQ9	**Felt overwhelmed?**	Not at all	No more than usual	Somewhat more than usual	Much more than usual
10 GHQ10	**Been satisfied with the way you have been carrying out your tasks?**	More than usual	Same as usual	Less than usual	Completely dissatisfied
11 GHQ11	**Been taking longer to do the things you do?**	Not at all	Same as usual	Longer than usual	Much longer than usual
12 GHQ12	**Been feeling exhausted and out of sorts?**	Not at all	No more than usual	Somewhat more than usual	Much more than usual
13 GHQ13	**Found it difficult to go back to sleep after waking up at night?**	Not at all	No more than usual	Somewhat more than usual	Much more than usual
14 GHQ14	**Been feeling the need to take medications to calm your nerves?**	Not at all	No more than usual	Somewhat more than usual	Much more than usual
15 GHQ15	**Been feeling anxious and tense all the time?**	Not at all	No more than usual	Somewhat more than usual	Much more than usual
16 GHQ16	**Been having hot or cold flashes?**	Not at all	No more than usual	Somewhat more than usual	Much more than usual
17 GHQ17	**Been getting scared or panicky for no good reason?**	Not at all	No more than usual	Somewhat more than usual	Much more than usual
18 GHQ18	**Lost much sleep over worry?**	Not at all	No more than usual	Somewhat more than usual	Much more than usual
19 GHQ19	**Felt worthless?**	Not at all	No more than usual	Somewhat more than usual	Much more than usual

20 GHQ20	**Found yourself wishing you were dead and free from it all?**	Not at all	No more than usual	Somewhat more than usual	Much more than usual
21 GHQ21	**Felt that life is completely hopeless?**	Not at all	No more than usual	Somewhat more than usual	Much more than usual
22 GHQ22	**Felt that life is not worth living?**	Not at all	No more than usual	Somewhat more than usual	Much more than usual
23 GHQ23	**Considered taking your own life?**	Definitely not	I do not think so	It has crossed my mind	Definitely yes
24 GHQ24	**Found that the idea of taking your own life kept coming to your mind?**	Definitely not	I do not think so	It has crossed my mind	Definitely yes
25 GHQ25	**Felt that you play a useful role in your environment?**	More than usual	Same as usual	Less than usual	Much less than usual
26 GHQ26	**Felt pleased with your actions in general?**	More than usual	Same as usual	Less than usual	Much less than usual
27 GHQ27	**Been able to enjoy your normal day-to-day activities?**	More than usual	Same as usual	Less than usual	Much less than usual
28 GHQ28	**Been managing to keep yourself busy with something useful, work?**	More than usual	Same as usual	Less than usual	Much less than usual

Drodzy Państwo, chcielibyśmy poznać Państwa podejście do pandemii COVID-19, ocenę wdrożonych procedur czerwonej strefy w całym kraju oraz jednocześnie ocenić jej wpływ na kondycję psychiczną.

Ankieta jest w pełni anonimowa, dobrowolna, na każdym etapie możliwa jest rezygnacja z udziału w badaniu. Czas trwania około 7 minut.

Skierowana do wszystkich ludzi mieszkających na terenie Polski powyżej 18rż.

1. Czy wyraża Pan(i) zgodę na udział w badaniu?
   Tak
   Nie

Sekcja socjidemograficzna

1. Płeć: Kobieta [ ] Mężczyzna [ ]
2. Wiek: ….. lat
3. Miejsce zamieszkania:
   a) Wieś
   b) Miasto <50tys.
   c) Miasto 50-250 tys.
   d) Miasto >250tys. mieszkańców
4. Wykształcenie:
   a) Podstawowe
   b) Gimnazjalne
   c) Zawodowe
   d) Średnie
   e) Wyższe niepełne
   f) Wyższe
5. Jaki jest Pana(i) stan cywilny:
   a) Zamężny/żonaty
   b) Rozwodnik
   c) Wdowa(iec)
   d) Związek partnerski
   e) Panna/Kawaler
6. Czy jest Pan(i) pracownikiem Ochrony zdrowia?

a) Tak
b) Nie

7. Czy w związku pandemią został(a) Pan(i) pozbawiony(a) możliwości zarobkowych?
   a) Tak, ale już wróciłe(a)m do pracy i moje zarobki nie uległy zmianie na gorsze
   b) Tak, wróciłe(a)m do pracy, lecz moje zarobki zmalały o nie więcej niż 25%
   c) Tak, wróciłe(a)m do pracy, lecz moje zarobki zmalały o więcej niż 25%
   d) Tak, zostałem całkowicie pozbawiony(a) dochodu
   e) Nie
   f) Zarówno przed jak i w trakcie pandemii nie pracowałem(am)
8. Czy w związku z pandemią korzystał(a) Pan(i) z usług psychiatry/psychologa?
   a) Tak
   b) Nie
9. Czy leczy się Pan z powodu schorzeń przewlekłych (np. nadciśnienie tętnicze, choroby serca, astma itp.)
   a) Tak
   b) Nie
10. Czy w przeszłości leczył(a) się Pan(i) psychiatrycznie?
   a) Tak
   b) Nie
11. Jeśli tak to z jakiego powodu?

Depresja/Zaburzenia lękowe/Zaburzenia snu/ Zaburzenia zachowania/ Choroby z kręgu schizofrenii/ Uzależnienia

12. Czy przyjmował(a) Pan(i) leki psychiatryczne (np. keterel, alprazolam, pramolan, etc.)
   a) Tak
   b) Nie
13. Czy w czasie pandemii COVID-19 został(a) Pan(i) objęta kwarantanną przez Stację Sanitarno-epidemiologiczną?
   a) Tak, jestem
   b) Tak, byłem
   c) Nie
14. Czy zdiagnozowano u Pana(i) COVID-19?
   a) Tak, jestem w trakcie choroby
   b) Tak jestem po chorobie
   c) Nie
15. Jeśli tak, to jaki był przebieg choroby?

Bezobjawowy/ Skąpoobjawowy (np. ból głowy, kaszel, gorączka <38stpC, pobyt w domu)/ Przebieg umiarkowany (temp. >38stp, duszność, bez hospitalizacji)/ Hospitalizacja

16. Czy według Pana(i) pandemia COVID-19 wpłynęła na ogólne Pana zdrowie psychiczne?
   a) Tak, zdecydowanie się pogorszyła
   b) Tak, pogorszyła się
   c) Nie
   d) Tak, uległa poprawie

Pracownik Ochrony Zdrowia

17. Jeśli tak to w jakim charakterze Pan(i) pracuje:

Lekarz/ Lekarz dentysta/pielęgniarstwo/ Ratownictwo medyczne/ Laborant/ Technik/ Inne

18. Czy został(a) Pan(i) oddelegowana przez dyrekcję lub rząd do pracy w oddziale COVIDOwym?
    a) Tak, rząd
    b) Tak, dyrekcja szpitala
    c) Nie
19. Czy pracował(a) Pan(i) bezpośrednio z pacjentami chorującymi na COVID-19?
    a) Tak, szpital jednoimienny
    b) Tak, oddział covidowy w szpitalu innym niż jednoimienny
    c) Tak, zarówno w szpitalu jak i w ramach POZ
    d) Podstawowa Opieka Zdrowotna
    e) Nie

Postawy

20. Jak ocenia Pan(i) dotychczasowe działania rządu w sprawie zwalczania COVID-19
    a) Bardzo dobrze
    b) Dobrze
    c) Ani dobrze, ani źle
    d) Źle
    e) Bardzo źle
21. Czy zgadza się Pan z działaniami podjętymi w Polsce (cały kraj objęty czerwoną strefą) w celu zwalczania pandemii COVID-19?
    a) Zdecydowanie się zgadza
    b) Zgadzam się
    c) Ani tak ani nie
    d) Nie zgadzam się
    e) Zdecydowanie się nie zgadzam
22. Czy od czasu zmiany zaleceń co do konieczności noszenia maseczek nosi Pan(i) maseczki na otwartej przestrzeni np. parki, place zabaw?
    a) Tak, zawsze
    b) Tak, zazwyczaj
    c) Nie
23. Czy od czasu zmiany zaleceń co do konieczności noszenia maseczek nosi Pan(i) maseczki w miejscach zamkniętych np. galerie handlowe, komunikacja?
    a) Tak, zawsze
    b) Tak, zazwyczaj
    c) Nie
24. W związku z pandemią COVID-19 unika Pan(i) spotkań z najbliższą rodziną?
    a) Zdecydowanie się zgadza
    b) Zgadzam się
    c) Nni tak ani nie
    d) Nie zgadzam się
    e) Zdecydowanie się nie zgadzam
25. W związku z pandemią COVID-19 unika Pan(i) spotkań z przyjaciółmi?
    a) Zdecydowanie się zgadza
    b) Zgadzam się
    c) Ani tak ani nie
    d) Nie zgadzam się
    e) Zdecydowanie się nie zgadzam
26. W związku z pandemią COVID-19 wyjścia z domu ograniczam do minimum (zakupy, praca itp.)?
    a) Zdecydowanie się zgadza
    b) Zgadzam się
    c) Ani tak ani nie

d) Nie zgadzam się
e) Zdecydowanie się nie zgadzam

27. Czy boi się Pani/Pan zachorowania na COVID-19?
a) Tak, w takim samym stopniu jak na inne choroby (np. serca)
b) Tak, ale w mniejszym stopniu jak na inne choroby (np. serca)
c) Tak, ale w większym stopniu jak na inne choroby (np. serca)
d) Nie, nie boję się w ogóle

28. W skali od 1 - 10 jak bardzo obawia się Pan(i) zachorowania na COVID-19? [……..]

29. W skali od 1 - 10 jak bardzo obawia się Pan(i) zachorowania na COVID-19 przez członków rodziny? …

30. Czy wyszukuje Pan(i) dodatkowych informacji odnośnie COVID-19 np. w internecie?
a) Tak
b) Nie

30. Czy śledzi Pan(i) codzienne statystki odnośnie zachorowań oraz śmiertelności z powodu COVID-19?
a) Tak
b) Nie

31. W celu zahamowania pandemii COVID-19 wszyscy powinniśmy zostać w domach?
a) Tak
b) Nie
c) Trudno powiedzieć

OGÓLNY KWESTIONARIUSZ ZDROWIA (GHQ-28)

Czy Pan(i) ostatnio:

1 GHQ1	**Czuje się dobrze i cieszy się dobrym zdrowiem?**	bardziej niż zwykle	tak jak zwykle	mniej niż zwykle	dużo mniej niż zwykle
2 GHQ2	**Czuje, że jest stale pod presją?**	zupełnie nie	nie bardziej niż zwykle	nieco bardziej niż zwykle	dużo bardziej niż zwykle
3 GHQ3	**Ma uczucie ucisku, napięcia w głowie?**	zupełnie nie	nie bardziej niż zwykle	nieco bardziej niż zwykle	dużo bardziej niż zwykle
4 GHQ4	**Ma bóle głowy?**	zupełnie nie	nie bardziej niż zwykle	nieco bardziej niż zwykle	dużo bardziej niż zwykle
5 GHQ5	**Czuje się zdolny(a) do podejmowania decyzji w różnych sprawach?**	bardziej niż zwykle	tak jak zwykle	mniej niż zwykle	zupełnie niezdolny
6 GHQ6	**Czuje, że czasami nie może nic robić ze względu na nerwy?**	zupełnie nie	nie bardziej niż zwykle	nieco bardziej niż zwykle	dużo bardziej niż zwykle
7 GHQ7	**Jest rozdrażniony(a) i w złym humorze?**	zupełnie nie	nie bardziej niż zwykle	nieco bardziej niż zwykle	dużo bardziej niż zwykle
8 GHQ8	**Czuje, że jest Pan(i) chory(a)?**	zupełnie nie	nie bardziej niż zwykle	nieco bardziej niż zwykle	dużo bardziej niż zwykle
9 GHQ9	**Czuje, że wszystko Pana(ią) przytłacza?**	zupełnie nie	nie bardziej niż zwykle	nieco bardziej niż zwykle	dużo bardziej niż zwykle
10 GHQ10	**Jest zadowolony(a) ze sposobu wykonywania swoich obowiązków?**	bardziej niż zwykle	tak jak zwykle	mniej niż zwykle	całkowicie niezadowolony
11 GHQ11	**Wykonywał(a) wszystkie czynności wolniej?**	zupełnie nie	tak jak zwykle	dłużej niż zwykle	dużo dłużej niż zwykle
12 GHQ12	**Czuje się wyczerpany(a), niedysponowany(a)?**	zupełnie nie	nie bardziej niż zwykle	nieco bardziej niż zwykle	dużo bardziej niż zwykle
13 GHQ13	**Ma kłopoty z ponownym zaśnięciem po przebudzeniu się ?**	zupełnie nie	nie bardziej niż zwykle	nieco bardziej niż zwykle	dużo bardziej niż zwykle

14 GHQ14	**Ma potrzebę zażywania środków uspokajających?**	zupełnie nie	nie bardziej niż zwykle	nieco bardziej niż zwykle	dużo bardziej niż zwykle
15 GHQ15	**Czuje się zdenerwowany(a) i napięty(a) przez cały czas?**	zupełnie nie	nie bardziej niż zwykle	nieco bardziej niż zwykle	dużo bardziej niż zwykle
16 GHQ16	**Czuje, że oblewa Pana(ią) gorąco lub zimno?**	zupełnie nie	nie bardziej niż zwykle	nieco bardziej niż zwykle	dużo bardziej niż zwykle
17 GHQ17	**Był(a) przestraszony(a) lub bliski(a) paniki bez wyraźnego powodu?**	zupełnie nie	nie bardziej niż zwykle	nieco bardziej niż zwykle	dużo bardziej niż zwykle
18 GHQ18	**Mniej spał(a) z powodu zamartwiania się?**	zupełnie nie	nie bardziej niż zwykle	nieco bardziej niż zwykle	dużo bardziej niż zwykle
19 GHQ19	**Uważa siebie za osobę bezwartościową?**	zupełnie nie	nie bardziej niż zwykle	nieco bardziej niż zwykle	dużo bardziej niż zwykle
20 GHQ20	**Pragnął/Pragnęła umrzeć i być daleko od wszystkich spraw?**	zupełnie nie	nie bardziej niż zwykle	nieco bardziej niż zwykle	dużo bardziej niż zwykle
21 GHQ21	**Ma poczucie, że życie jest beznadziejne?**	zupełnie nie	nie bardziej niż zwykle	nieco bardziej niż zwykle	dużo bardziej niż zwykle
22 GHQ22	**Myślał(a), że nie warto żyć?**	zupełnie nie	nie bardziej niż zwykle	nieco bardziej niż zwykle	dużo bardziej niż zwykle
23 GHQ23	**Brał(a) pod uwagę możliwość odebrania sobie życia ?**	stanowczo nie	nie sądzę	przeszło mi przez myśl	stanowczo tak
24 GHQ24	**Zauważył(a), że przychodzi Panu(i) do głowy myśl o odebraniu sobie życia ?**	stanowczo nie	nie sądzę	przeszło mi przez myśl	stanowczo tak
25 GHQ25	**Ma poczucie, że odgrywa znaczącą rolę w otoczeniu?**	bardziej niż zwykle	tak jak zwykle	mniejszą niż zwykle	Znacznie mniejszą
26 GHQ26	**Jest ogólnie zadowolony(a) ze swoich działań ?**	bardziej niż zwykle	tak jak zwykle	mniej niż zwykle	dużo mniej niż zwykle
27 GHQ27	**Potrafi cieszyć się zwykłymi codziennymi zajęciami?**	bardziej niż zwykle	tak jak zwykle	mniej niż zwykle	dużo mniej niż zwykle
28 GHQ28	**Potrafił(a) wypełnić sobie czas pożytecznymi zajęciami, pracą?**	bardziej niż zwykle	tak jak zwykle	mniej niż zwykle	dużo mniej niż zwykle
